# Label-Free Quantitation and Mapping of the ErbB2 Tumor Receptor by Multiple Protease Digestion with Data-Dependent (MS1) and Data-Independent (MS2) Acquisitions

**DOI:** 10.1155/2013/791985

**Published:** 2013-04-04

**Authors:** Jason M. Held, Birgit Schilling, Alexandria K. D'Souza, Tara Srinivasan, Jessica B. Behring, Dylan J. Sorensen, Christopher C. Benz, Bradford W. Gibson

**Affiliations:** ^1^The Buck Institute for Research on Aging, 8001 Redwood Boulevard, Novato, CA 94945, USA; ^2^Department of Medicine and Division of Oncology-Hematology, University of California, San Francisco, CA 94143, USA; ^3^Department of Pharmaceutical Chemistry, University of California, San Francisco, CA 94143, USA

## Abstract

The receptor tyrosine kinase ErbB2 is a breast cancer biomarker whose posttranslational modifications (PTMs) are a key indicator of its activation. Quantifying the expression and PTMs of biomarkers such as ErbB2 by selected reaction monitoring (SRM) mass spectrometry has several limitations, including minimal coverage and extensive assay development time. Therefore, we assessed the utility of two high resolution, full scan mass spectrometry approaches, MS1 Filtering and SWATH MS2, for targeted ErbB2 proteomics. Endogenous ErbB2 immunoprecipitated from SK-BR-3 cells was in-gel digested with trypsin, chymotrypsin, Asp-N, or trypsin plus Asp-N in triplicate. Data-dependent acquisition with an AB SCIEX TripleTOF 5600 and MS1 Filtering data processing was used to assess peptide and PTM coverage as well as the reproducibility of enzyme digestion. Data-independent acquisition (SWATH) was also performed for MS2 quantitation. MS1 Filtering and SWATH MS2 allow quantitation of all detected analytes after acquisition, enabling the use of multiple proteases for quantitative assessment of target proteins. Combining high resolution proteomics with multiprotease digestion enabled quantitative mapping of ErbB2 with excellent reproducibility, improved amino acid sequence and PTM coverage, and decreased assay development time compared to typical SRM assays. These results demonstrate that high resolution quantitative proteomic approaches are an effective tool for targeted biomarker quantitation.

## 1. Introduction

Large-scale efforts to understand biological processes, such as functional genomics, systems biology, and cancer mutation analysis, continue to uncover master regulators of cell signaling and potential biomarkers of human disease [1–3]. Understanding the regulation of these biomarkers and validating their role in disease processes, however, depends on measurement of their expression and regulatory status in response to different cellular conditions, drug treatments, or patient samples. The receptor tyrosine kinase ErbB2 (HER2) is an important biomarker that is overexpressed in ~25% of all breast cancers, is a key drug target, and is a member of a biologically important family of tyrosine kinases. ErbB2 is known to be heavily regulated by posttranslational modifications (PTMs) which can modulate its kinase activity and protein-protein interaction partners [4–6]. ErbB2 is also subject to membrane-associated proteolytic processing and has several poorly understood isoform variants [7].

Mass spectrometry-based proteomics combined with stable-isotope labeling or tagging is a powerful technique for large-scale quantitation and unbiased characterization of the proteome [8, 9]. Nonetheless, it is well known that unbiased discovery proteomics typically suffers from limited dynamic range and sampling efficiency, which can only be partially addressed by incorporating orthogonal fractionation steps. Alternatively, if one is interested in targeting a small subset of the proteome, selected reaction monitoring (SRM) mass spectrometry is often employed due to its improved dynamic range, reproducibility, and sensitivity [10]. Coupling immunoprecipitation with SRM analysis is a particularly useful combination for the analysis of proteins of interest [11, 12]. However, SRM requires significant upfront assay development time to develop specific SRM transitions and, even with multiplexing and/or retention time scheduling, only a limited number (≤150) of target peptide analytes can be measured in a single LC-MS analysis. SRM also acquires a small, predefined subset of analyte information in a sample run that cannot be mined after acquisition based on new ideas or hypotheses.

Recent breakthroughs using high-resolution quantitative proteomics have emerged as powerful alternatives to SRM analysis that can be performed on many of the same mass spectrometer platforms that are also optimum for discovery-type mass spectrometry experiments [13]. These include approaches for label-free quantitation based on MS1 precursor ion intensity measurements [14, 15]. Recently, we reported a method based on extracting ion intensity data from the MS1 scans, MS1 Filtering, in a platform-independent manner using the Skyline environment and then applied this method for various data-dependent mass spectrometry acquisitions [16]. As Skyline was originally developed for SRM experiments, MS1 Filtering uses many of the same tools to facilitate quantitation of the peptide precursors, although in this case all peptides identified in discovery-type data-dependent acquisitions, providing information beyond simple peptide identifications. However, since the quantitation is performed at the MS1 level, site determination of PTMs of interest cannot be resolved in all cases by MS1 Filtering alone. Alternatively, a data-independent acquisition approach, SWATH MS2, cycles through consecutive 25 *m/z* precursor isolation windows (swaths) collecting fragment ion spectra for all detectable analytes within a sample [17, 18]. Notably, SWATH MS2 acquisitions can be used to confirm and quantify specific PTMs with the acquired MS2 peptide fragmentation data. 

Most SRM assays are developed for trypsin-digested target proteins because trypsin is assumed to be the most consistent and reproducible protease for protein digestion [19]. However, use of a single protease limits both amino acid coverage and PTM detection of a protein of interest because proteolysis with a single enzyme produces only a subset of the potential peptides that can be detected by LC-MS [20]. Due to the significant assay development time and the limited number of analytes measurable by SRM, there has been very little exploration of the application of other proteases or double digestions, trypsin plus a second enzyme, for targeted proteomics. In addition, there have been few reports of targeted SRM-based assays using less specific enzymes, such as chymotrypsin, even though these proteases can significantly enhance amino acid and PTM coverage of target proteins [21]. 

High resolution quantitative proteomics approaches such as MS1 Filtering and SWATH MS2 analysis have comparable reproducibility and dynamic range as SRM [5, 16] but have the advantage that they require little to no assay development time and can quantify all detectable analytes in a sample after acquisition. Therefore, while these approaches are not of high throughput or large scale, they are ideally suited for label-free quantitative mapping of target proteins such as ErbB2 using multiple proteases. In this study, we analyzed endogenous ErbB2 immunoprecipitated from SK-BR-3 cell lysates which was in-gel digested in triplicate with trypsin, Asp-N, and chymotrypsin or double digested with trypsin plus Asp-N. The application of MS1 Filtering for data-dependent acquisition and additional SWATH MS2 workflows enabled quantitation of each of the 60-140 ErbB2 peptides generated per digestion condition, which facilitated for the first time the assessment of the reproducibility of these protease conditions for targeted proteomics. 

## 2. Materials and Methods

### 2.1. Materials

Anti-c-ErbB2/c-Neu (Ab-3) mouse (3B5) antibody was purchased from Calbiochem. Protein G Sepharose 4 Fast Flow was from GE Healthcare. SDS-PAGE 4%–12% gels and SDS-PAGE loading buffer were from Invitrogen. Sequencing grade trypsin was from Promega. Asp-N, chymotrypsin, and Complete Protease Inhibitors (EDTA free) were from Roche. C18 zip tips were from Millipore. HPLC solvents including acetonitrile and water were obtained from Burdick & Jackson. Reagents for protein chemistry including N-ethylmaleimide, dithiothreitol (DTT), ammonium bicarbonate, and formic acid were purchased from Sigma Aldrich.

### 2.2. Cell Culture and Immunoprecipitation

SK-BR-3 cells were obtained from American Type Culture Collection (ATCC) and grown under ATCC-recommended culture conditions, DMEM plus 10% fetal bovine serum. Four 15 cm plates of SK-BR-3 cells were lysed with 750 *μ*L ice cold lysis buffer (50 mM HEPES, 100 mM NaCl, 1% NP-40, 0.01% SDS, 1% sodium deoxycholate, 1 mM NEM, and Complete Protease Inhibitors). To immunopurify ErbB2 from each plate of cells, 2.5 *μ*g of ErbB2 (Ab-3) antibody was added for 1 hr with rotation at 4°C. A 15 *μ*L Protein G resin was then added and incubated overnight at 4°C. Beads were washed four times with cold lysis buffer for 10 min before addition of reducing SDS-PAGE loading buffer. Samples were pooled into a single sample prior to SDS-PAGE.

### 2.3. In-Gel Digestion

Protein bands of interest were manually excised out of the gel, destained and dehydrated with acetonitrile, reduced with 10 mM DTT (56°C, 1 hr), and alkylated with 55 mM N-ethylmaleimide (25°C, 45 min). Prior to enzymatic digestion, excess reagents were removed and the gel pieces were washed twice with 25 mM ammonium bicarbonate and dehydrated by vacuum centrifugation. For digestion, gel samples were incubated with either 250 ng sequencing grade trypsin, Asp-N, or chymotrypsin (37°C overnight). For the trypsin plus Asp-N double digest, overnight trypsin digestion was followed by dehydration by vacuum centrifugation and subsequent addition of 250 ng Asp-N (37°C overnight). Peptides were extracted from the gel with 100 *μ*L water, and twice with 50% ACN/5% formic acid with 10 min of sonication and 10 min vortexing per extraction. Samples were vacuum centrifuged to remove ACN, acidified with formic acid, and C18 zip-tipped prior to mass spectrometry. 

### 2.4. Mass Spectrometric and Chromatographic Methods and Instrumentation

Samples were analyzed by reverse-phase HPLC-ESI-MS/MS using an Eksigent Ultra Plus nano-LC 2D HPLC system connected to a quadrupole time-of-flight TripleTOF 5600 mass spectrometer (AB SCIEX). Details for the mass spectrometric and chromatographic methods are described in detail in the Supplementary Methods (See Supplementary Material available online at http://dx.doi.org/10.1155/2013/791985). Briefly, samples were acquired in *data-dependent mode* on the TripleTOF 5600 to obtain MS/MS spectra for the 30 most abundant parent ions following each survey MS1 scan. Additional data sets were recorded in *data-independent mode* using SWATH MS2 acquisitions. In the SWATH MS2 acquisition, instead of the Q1 quadrupole transmitting a narrow mass range through to the collision cell, a wider window of ~25 *m/z* is passed in incremental steps over the full mass range 400–1000 *m/z* (for full details see Supplemental Methods).

### 2.5. Bioinformatic Database Searches

Mass spectrometric data was searched using Mascot [22] server version 2.3.02. Peak lists for Mascot searches were generated using the AB SCIEX MGF converter version 1.2.0.193. MS/MS datasets were also analyzed using the database search engine ProteinPilot [23] (AB SCIEX Beta 4.1.46, revision 460) with the Paragon algorithm (4.0.0.0, 459). All details regarding search parameters, fixed and variable modifications, enzyme specificity, databases used, scoring, false discovery rate analysis (FDR) are described in the Supplementary Methods. Peptide FDR rate was set to 5% or less based on decoy database searching and all peptides included for analysis had a score representing ≤1% FDR in at least one of the search engine results. PTM site assignment was initially suggested by search engines ProteinPilot and Mascot (for details see below) and confirmed by manual inspection using previously defined criteria [24]. 

### 2.6. Quantitative Skyline MS1 Filtering Analysis

MS1 chromatogram-based quantitation was performed in Skyline [25] (http://proteome.gs.washington.edu/software/skyline/). Details for MS1 Filtering and MS1 ion intensity chromatogram processing in Skyline were described recently in detail by Schilling et al. [16]. Briefly, comprehensive spectral libraries were generated in Skyline using the BiblioSpec algorithm [26] from database searches of the raw data files prior to MS1 Filtering. Subsequently, raw files acquired in data-dependent mode were directly imported into Skyline 1.3 and MS1 precursor ions extracted for all peptides present in the MS/MS spectral libraries. Quantitative analysis is based on extracted ion chromatograms (XICs) and resulting precursor ion peak areas for each peptide M, M+1, and M+2, the first, second, and third isotope peak of the isotopic envelope. 

### 2.7. Quantitative SWATH Data Analysis in Skyline

Datasets from SWATH MS2 acquisitions were processed using the full scan MS/MS filtering module for data-independent acquisition within Skyline 1.3. The top 8 fragment ions were extracted from SWATH MS2 acquisitions within Skyline using a fragment ion resolution setting of 10,000. 

### 2.8. Statistical Analysis

Two-sample comparison of means was used to estimate the fold change significantly detectable (*P* ≤ 0.05) based on %CV between two conditions for three biological replicates per sample. Two-sample comparison of means is a statistical test that can be used to determine the statistical likelihood of detecting a given difference between two samples with a defined sample size, means, and standard of deviations for each sample. Calculations were determined using Stata 10 (StataCorp) with an alpha of 0.05 and power of 0.8.

## 3. Results

**Figure 1:**
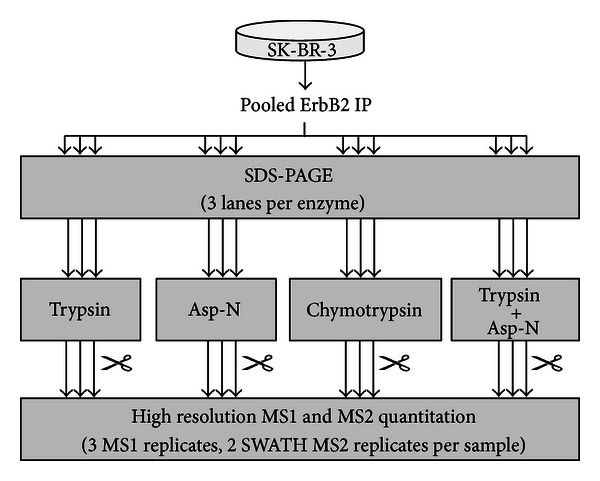
Workflow for ErbB2 targeted proteomics using multiprotease digestion and high resolution mass spectrometry quantitation. ErbB2 immunopurified from four 15 cm plates of untreated SK-BR-3 cells was pooled into a single sample. The sample was split into 12 aliquots and separated by SDS-PAGE. Triplicate in-gel digestion was performed using either trypsin, Asp-N, chymotrypsin, or a double digestion with trypsin plus Asp-N. Each sample was analyzed using an AB SCIEX TripleTOF 5600 mass spectrometer. Two approaches for high resolution LC-MS/MS quantitation were employed, MS1 Filtering and SWATH MS2 acquisition.

 The workflow in Figure 1 was developed to assess the utility of MS1 Filtering and SWATH MS2 for the multiprotease digestion of ErbB2. To eliminate biological variability, endogenous ErbB2 immunoprecipitated from human SK-BR-3 cells was pooled into a single sample. SDS-PAGE was used to isolate ErbB2 from the antibody, protein G, and most protein-protein interaction partners in the immunoprecipitate. ErbB2 was in-gel digested in triplicate with either trypsin, Asp-N, or chymotrypsin individually or double digested with trypsin plus Asp-N. Samples were analyzed using an AB SCIEX TripleTOF 5600 hybrid quadrupole time-of-flight mass spectrometer with data-dependent acquisitions to identify peptides. For each sample, three replicate mass spectrometry analyses were acquired for MS1 Filtering processing as well as two SWATH MS2 acquisitions. 

**Figure 2:**
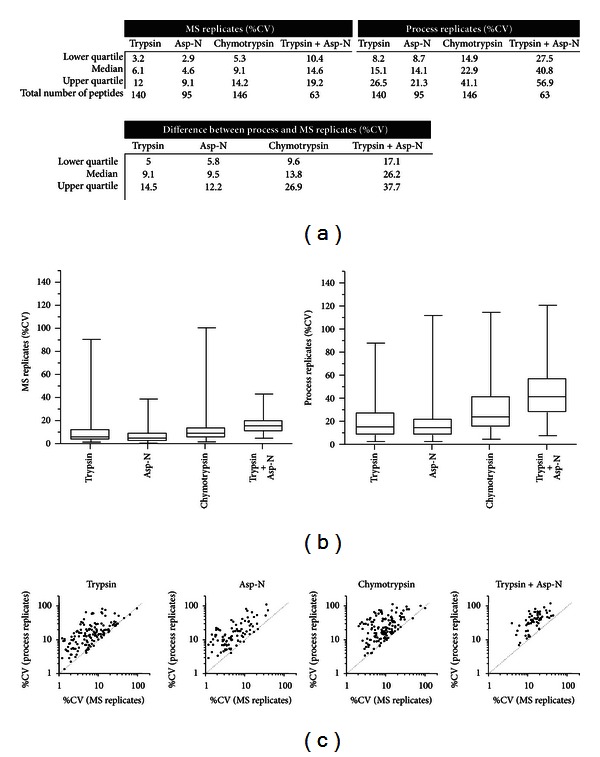
Reproducibility assessment of MS1 Filtering and digestion of ErbB2 by multiple proteases. (a) Percent coefficient of variation (%CV) of individual ErbB2 peptide samples was determined by MS1 Filtering. Each individually digested sample was analyzed with data-dependent acquisition in triplicate, MS replicates, and each enzyme digestion was performed in triplicate, process replicates. The difference between process and MS replicates represents the added peptide variability due to enzyme digestion. (b) Box and whisker plot of the %CV of MS and process replicates for ErbB2 peptides detected in the four enzyme conditions assessed. (c) Scatter plot of the MS replicate and process replicate %CV for each ErbB2 peptide detected.

 All identified ErbB2 peptides were imported into Skyline for each digestion condition and corresponding spectral libraries were made with no filtering for the types of modifications or cleavage sites of the peptides. The number of peptides identified for ErbB2 ranged from 63 (trypsin plus Asp-N) to 146 peptides (chymotrypsin) (Figure 2(a)). The coverage with trypsin plus Asp-N was likely the lowest due to the decreased average size of the peptides generated which limits their detection by LC-MS. The entire list of ErbB2 peptides is listed in Supplementary Table 1. Data-dependent and SWATH MS2 acquisitions were independently imported into separate Skyline documents for peak integration based on the retention time of the MS/MS spectra of each identified peptide. The percent coefficient of variation (%CV), the standard of deviation divided by the mean, was determined for each precursor or fragment ion for MS1 Filtering and SWATH MS2, respectively.

 To assess the reproducibility of the LC-MS analysis alone, the %CV of each peptide precursor in each individual ErbB2 sample was determined by MS1 Filtering for the three replicate data-dependent mass spectrometry acquisitions (Figure 2(a)). The %CV of these MS replicates was below 20% for more than 75% of the peptides identified in each of the four enzyme conditions. Therefore, the technical mass spectrometry reproducibility of high resolution MS1 Filtering analysis is on par with SRM analysis (Figure 2(b)). To quantify the reproducibility of digestion, the %CV across the triplicate digestion conditions was determined for each enzyme (Figures 2(a) and 2(b)). These process replicate %CVs were the best for trypsin and Asp-N with a median %CV of 15.1% and 14.1%, respectively, with an additional variability of only 9.1% and 9.5% more than the MS replicates for each enzyme. While the process replicate %CVs for chymotrypsin were significantly higher than trypsin (*P* < 0.001), the median %CV of the process chymotrypsin replicates was 13.8% higher than the MS replicates alone, comparable to trypsin and Asp-N individually. In contrast, the median %CV for the double digestion (trypsin plus Asp-N) process replicates was 26.2% higher than the MS replicates. These results suggest that digestion with a single protease, even using less specific proteases such as chymotrypsin, is far more reproducible than a double digestion using two relatively specific, consistent enzymes. Overall, there was no apparent correlation between process variation and MS variation (Figure 2(c)).

 Peptide properties such as cleavage specificity and the number of missed cleavages are often assumed to influence the reproducibility of peptide generation by proteases [27, 28]. For example, peptides with several missed cleavages are often considered less ideal candidates for quantitation since it is assumed that a protease will not partially cleave consistently [19]. An additional consideration is whether the cleavage site has two or more potential cleavage sites in a row, also known as “ragged ends” [29]. This is because trypsin and potentially other enzymes used for sequencing do not efficiently cleave off a C-terminal lysine or arginine even if the penultimate residue is also a cleavage site; that is, they exhibit poor exopeptidase activity. However, these assumptions have been largely left untested due to the difficulty of developing SRM assays to a large, representative population of peptides in a target protein needed for a comprehensive evaluation of these parameters. However, the application of MS1 Filtering and SWATH MS2 can overcome these limitations and enable analysis of these parameters on the reproducibility of peptide generation.

**Figure 3:**
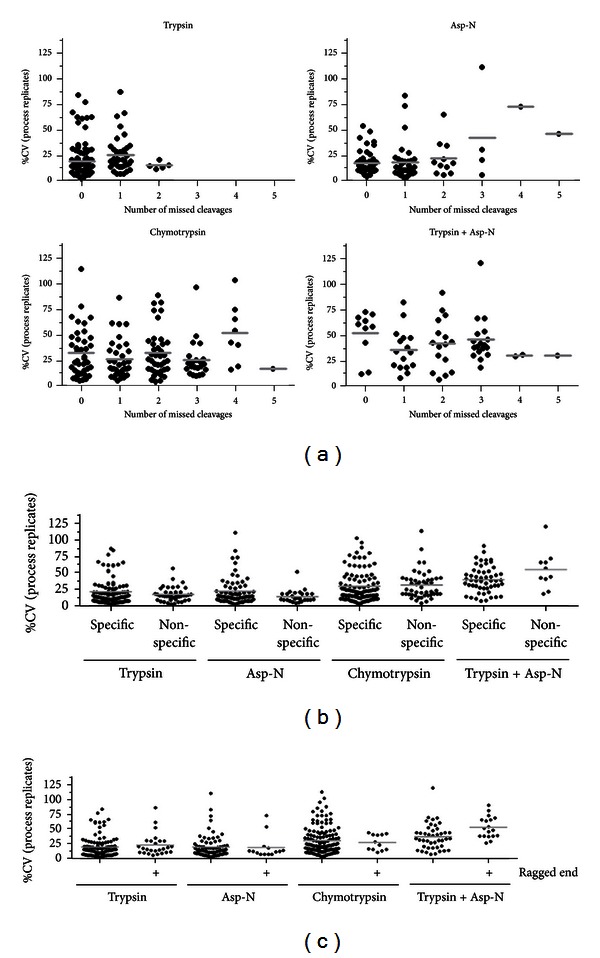
Assessing the impact of nonspecific cleavage, missed cleavages, and ragged ends on the reproducibility of ErbB2 peptides. The %CV for all ErbB2 peptides detected in each of the four enzyme conditions tested based on (a) number of missed cleavages, (b) specificity of cleavage, and (c) ragged ends. Peptides with at least one nonspecific cleavage or ragged end were considered nonspecific or ragged end peptides. Grey lines indicate the median value for each condition.

 We determined the influence of cleavage specificity, number of missed cleavages, and presence of ragged ends on the reproducibility of ErbB2 peptide generation by assessing the %CV of the process replicates using MS1 Filtering. Trypsin typically generated peptides with 0-1 missed cleavages, Asp-N generated peptides with predominantly 0–2 missed cleavages, and chymotrypsin and the double trypsin plus Asp-N digestion peptides typically had 0–3 missed cleavages (Figure 3(a)). However, an increased number of missed cleavages within these ranges did not decrease peptide reproducibility, suggesting that while these proteases may not cleave to completion, they have consistent, reproducible substrate specificity (Figure 3(a)). We also examined the effect of nonspecific cleavage and ragged ends on peptide reproducibility, though neither parameter had a significant impact on reproducibility (Figures 3(b) and 3(c)). These results indicate that many of the assumptions regarding the ideal peptide parameters for maximal reproducibility for quantitative proteomics are incorrect and difficult to predict. Rather, an important step to maximize quantitative mapping of a target protein is empirical assessment of peptide reproducibility and selection of robust peptides for quantitation based on experimental results.

**Figure 4:**
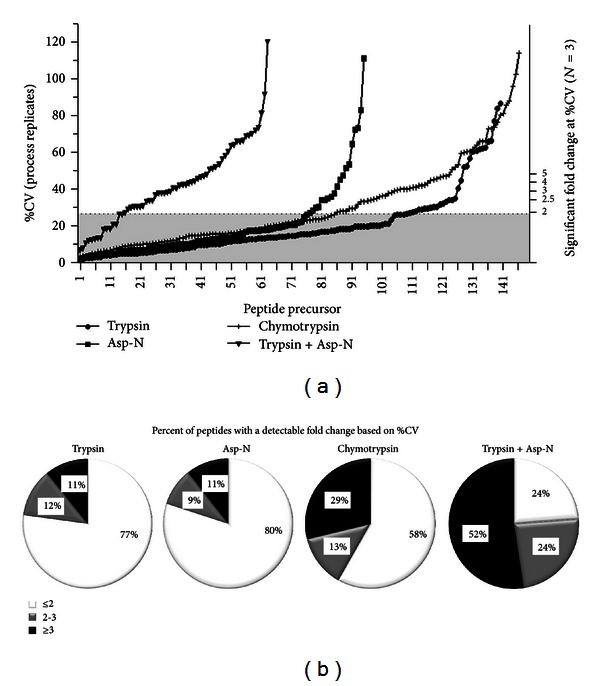
ErbB2 peptides that can reproducibly detect a given fold change using high resolution mass spectrometry and MS1 Filtering. (a) ErbB2 peptides from each of the four digestion conditions tested rank ordered by %CV. The left *y*-axis represents the %CV for each peptide and the right *y*-axis represents the detectable fold change between two conditions for three biological replicates per condition as determined by a two-sample comparison of means test. A twofold change can be detected by peptides with a %CV less than 27% which are shaded. (b) The percent of all peptides identified (5% FDR) in each of the four digestion conditions that can detect a 2-fold change or less (≤2), a 2-3-fold change (2-3), or only a fold change greater than 3 (≥3) between two conditions in three biological replicates per condition.

 Maximizing the quantifiable sequence coverage and PTM status of important biomarkers such as ErbB2 is critical for in-depth assessment of protein and isoform expression, regulatory and activation status, and proteolytic processing. Based on the empirically determined process reproducibility, which is the %CV of all peptides detected, the assessable sequence coverage of a target protein can be estimated for a fixed number of biological replicates and fold change detectable between conditions. Two-sample comparison of means estimates that a 27% CV can detect a significant twofold change between conditions with three biological replicates, a typical fold change cutoff used in quantitative proteomics studies. Peptides that are rank ordered by %CV for each digestion are shown in Figure 4(a), with the fold change detectable for three biological replicates shown on the right *y*-axis. These results suggest that over 75% of the peptides identified in ErbB2 samples digested by trypsin or by Asp-N and 58% of chymotryptic peptides can quantify a twofold change between two conditions (Figure 4(b)). Nearly 90% of peptides digested by trypsin or by Asp-N and 70% of chymotryptic peptides can detect a 3-fold change between conditions. The double trypsin plus Asp-N digestion is less effective than anticipated based on the initial %CV assessment, as described above. 

**Figure 5:**
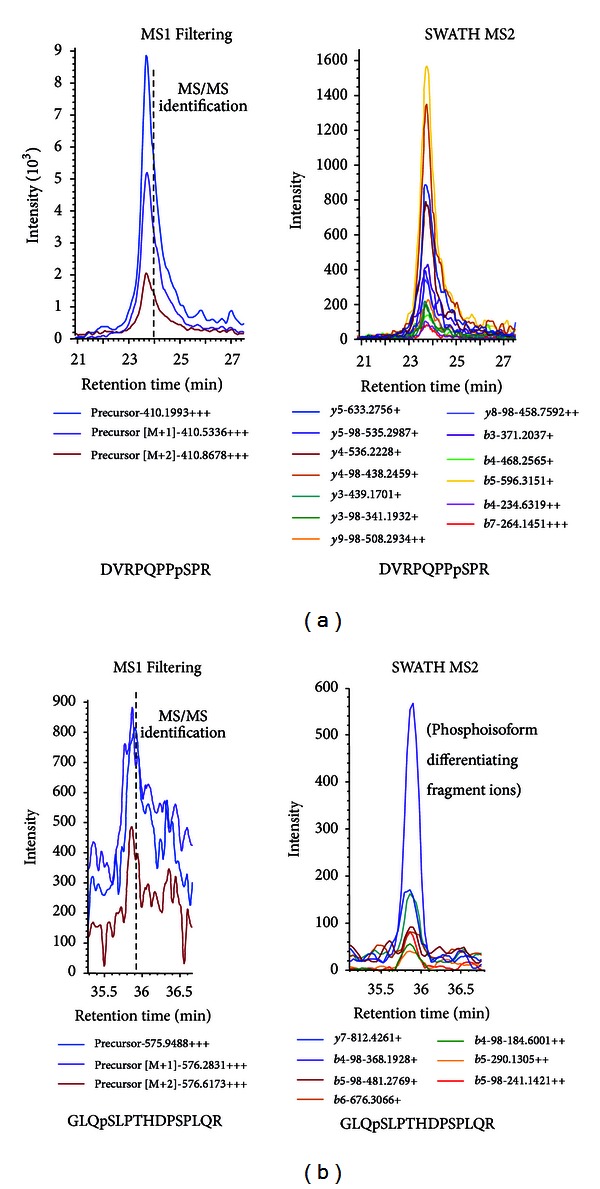
Comparison of high resolution extracted ion chromatograms by MS1 Filtering and SWATH MS2 for the phosphorylated ErbB2 peptides DVRPQPPpSPR and GLQpSLPTHDPSPLQR. (a) MS1 Filtering is applied to the MS1 scan of data-dependent high resolution LC-MS/MS analyses. MS1 Filtering can be used to extract the ion chromatogram of the monoisotopic precursor as well as the first and second naturally occurring isotopes, [M+1] and [M+2], respectively, as shown for the ErbB2 phosphopeptide DVRPQPPpSPR. Data-independent SWATH MS2 acquisitions complement MS1 Filtering by acquiring fragment ion intensities from MS2 scans which can also be used for quantitation. (b) Since the precursor is intact, MS1 Filtering cannot differentiate between multiple potential phosphoisoforms of the ErbB2 peptide GLQpSLPTHDPSPLQR from GLQSLPpTHDPSPLQR and GLQSLPTHDPpSPLQR based on mass. SWATH MS2 acquires the MS/MS fragment ions of the peptides detected and can be reconstructed after acquisition to confirm the site of modification. Fragment ions y7, b4-98, b5-98, b6, b4-98^2+^, b5^2+^, and b5-98^2+^ are all specific to the phosphoisoform GLQpSLPTHDPSPLQR.

 SWATH MS2 acquisitions can complement data-dependent acquisition and MS1 Filtering particularly for the analysis of PTM peptides.  Figure 5(a) compares the typical results from MS1 Filtering and SWATH MS2 for the ErbB2 phosphopeptide DVRPQPPpSPR. MS1 Filtering can be used to quantify data-dependent acquisitions in which the MS/MS identification is made, whereas a second acquisition using SWATH MS2 allows quantitation of the fragment ions of a peptide at the MS2 level. Confirming the posttranslationally modified residue is a critical step in protein PTM analysis; however, since MS1 Filtering cannot differentiate between different sites of modification on a peptide should more than one possibility exist, SWATH MS2 plays an important role in PTM quantitation of a target protein. For example, Figure 5(b) shows the extracted ion chromatograms from MS1 Filtering for the triply charged peptide GLQpSLPTHDPSPLQR which is unable to differentiate between potential phosphoisoforms of this peptide. With SWATH MS2 acquisition and processing, specific or unique fragment ions that differentiate between phosphoisoforms can be extracted for quantitation and confirm the modification site. If only a single phosphoisoform is detectable, the most intense fragment ion was chosen for quantitation. In total, eight phosphorylation sites and one acetylation site were identified in the ErbB2 immunopurified from untreated SK-BR-3 cells with the peptide sequences, %CV of SWATH acquisitions, as well as precursor and fragment ion information listed in Table 1. 

## 4. Discussion

**Figure 6:**
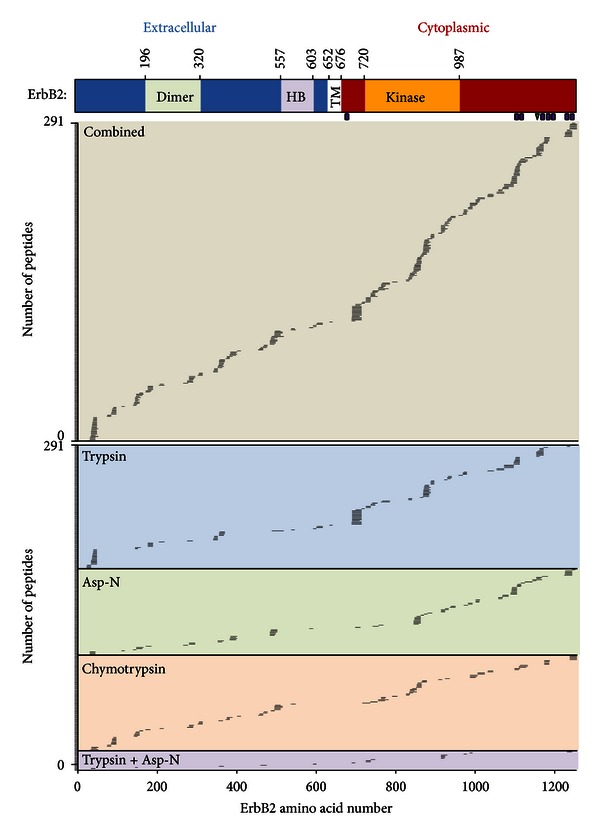
Coverage map of ErbB2 peptides that can significantly detect a twofold change between conditions by high resolution proteomics. ErbB2 has an N-terminal extracellular domain (1-652) which includes a dimerization (dimer) and herceptin binding (HB) domain. In addition, ErbB2 has a transmembrane domain (TM) as well as a C-terminal cytoplasmic domain which contains its kinase domain. Sites of ErbB2 phosphorylation (purple rectangles) and acetylation (green triangles) identified in this study are indicated. The 291 peptides estimated to be able to detect a significant twofold change between conditions (%CV ≤ 27 by MS1 Filtering) from all four digestion conditions were ordered beginning by amino acid to demonstrate the coverage of ErbB2 quantifiable by high resolution proteomics. The peptide coverage for each individual digestion condition is also indicated.

The combination of multiprotease enzyme digestion with high resolution, full scan quantitative proteomics approaches such as MS1 Filtering and SWATH MS2 acquisition is an effective and viable alternative to SRM analysis for targeted proteomics. In this study, we quantified 444 ErbB2 peptide precursors and found that 291 were sufficiently reproducible to detect a twofold change between two conditions. This corresponds to 63.7% of the ErbB2 protein sequence and 799 of 1255 amino acids (Figure 6). The application of MS1 Filtering and SWATH MS2 to targeted proteomics using even a single enzyme, such as trypsin, can vastly improve assay throughput, decrease assay development time, and increase the breadth of the sequence coverage and PTMs that can be quantified. As demonstrated in this study, MS1 Filtering and SWATH MS2 were used to quantify 140 tryptic ErbB2 peptides, typically beyond the scope of peptide SRM assays, corresponding to 435 ErbB2 amino acids and a sequence coverage of 35%. In addition, these analyses can be performed on a single mass spectrometer without any assay development time. Since digestions of immunoprecipitated proteins have limited sample complexity, it may be possible to combine multiprotease digestions of a target protein into a single sample to improve sample acquisition throughput for the analysis of multiple conditions. While this study was based on in-gel digestion, multiprotease digestions in solution could be used to improve sample throughput.

In conclusion, our study demonstrates that data-dependent (MS1) and data-independent (MS2) acquisition are both powerful tools for the analysis of target proteins and complement SRM-based assays. One specific advantage is that, unlike SRM, data for all detectable analytes is acquired and can be mined after acquisition. Therefore, MS1 Filtering and SWATH MS2 methods are ideal for the analysis of samples where material is limited and/or stability may be a factor since the data can be subsequently reanalyzed if there is a change in hypotheses or a new result points to different PTMs to be investigated. In addition, MS1 Filtering and SWATH MS2 can in principle perform absolute quantitation, much like SRM, when stable isotope-labeled peptides are spiked in at known concentrations. While SRM assays are ultimately the most sensitive assays for clinical samples, high resolution proteomic approaches such as MS1 Filtering and SWATH MS2 can facilitate SRM assay development by filtering a large list of identified candidate peptides for further analysis. Lastly, future validation of MS1 Filtering and SWATH MS2 for clinical sample analysis may provide alternate quantitative approaches to SRM for the analysis of challenging peptide analytes. 

## 5. Conclusions

Combining high resolution data-dependent (MS1) and data-independent (MS2) mass spectrometry with multiprotease digestion of target proteins greatly improves quantitation coverage and is an effective alternative to SRM-based assays for targeted proteomics. 

## Supplementary Material

Supplementary Material: includes the results from Skyline for the ErbB2 peptides detected and quantified in this study.

## Figures and Tables

**Table 1:** Phosphorylated and acetylated ErbB2 peptides identified and quantified by SWATH MS2. Modifications include phosphorylation [+80], acetylation [+42], and oxidation [+16].

Peptide	SWATH %CV	Modified residue	Enzyme	*z*	Fragment ion
DPPERGAPPSTFKGT[+80]PTA	15.9%	1240	Asp-N	3	b7
DVRPQPPS[+80]PR	10.9%	1151	Asp-N	3	b5
EGPLPAARPAGAT[+80]LERPK	12.4%	1166	Trypsin	2	y14
ERPKTLS[+80]PGKNGVVK	24.8%	1174	Asp-N	4	y4
GAPPSTFKGT[+80]PTA	25.1%	1240	Trypsin + Asp-N	2	y3
GLQS[+80]LPTHDPSPLQR	26.6%	1100	Trypsin	3	b4
K[+42]GTPTAENPEYLGLDVPV	23.7%	1238	Chymotrypsin	2	b11
KGT[+80]PTAENPEYLGLDVPV	18.5%	1240	Chymotrypsin	3	b8
LLQETELVEPLT[+80]PSGAM[+16]PNQAQM[+16]R	22.9%	701	Trypsin + Asp-N	3	y12
LLQETELVEPLT[+80]PSGAM[+16]PNQAQMR	30.1%	701	Trypsin	3	b8
LLQETELVEPLT[+80]PSGAMPNQAQM[+16]R	30.8%	701	Trypsin	3	b8
LLQETELVEPLT[+80]PSGAM[+16]PNQAQM[+16]R	20.4%	701	Trypsin	3	y12
PAGAT[+80]LERPK	18.7%	1166	Trypsin	2	y6
S[+80]GGGDLTLGLEPSEEEAPR	30.8%	1154	Trypsin	3	y8
SPLAPSEGAGS[+80]DVFDGDLGM[+16]GAAK	54.5%	1082	Trypsin	3	y10
TLS[+80]PGKNGVVK	18.9%	1174	Trypsin	2	y9
