# Supplementary material for: Label-Free Quantitation and Mapping of the ErbB2 Tumor Receptor by Multiple Protease Digestion with Data-Dependent (MS1) and Data-Independent (MS2) Acquisitions
Source: Int J Proteomics. 2013 Apr 4;2013:791985. doi: 10.1155/2013/791985 (PMC3654371; doi:10.1155/2013/791985)
Supplement: Supplementary file 1 — Supplementary Material: includes the results from Skyline for the ErbB2 peptides detected and quantified in this study. [file 791985.f1.docx]

**Supplemental Methods S1:**

*Mass spectrometric and chromatographic methods and instrumentation –*

AB SCIEX TripleTOF 5600: Samples were analyzed by reverse-phase HPLC-ESI-MS/MS using an Eksigent Ultra Plus nano-LC 2D HPLC system (Dublin, CA) which was directly connected to an AB SCIEX quadrupole time-of-flight (QqTOF) TripleTOF 5600 mass spectrometer (AB SCIEX, Concord, CAN) in direct injection mode. The autosampler was operated in full injection mode overfilling a 1 µl loop with 3 µl analyte for optimal sample delivery reproducibility. Briefly, after injection, peptide mixtures were transferred onto the analytical C18-nanocapillary HPLC column (C18 Acclaim PepMap100, 75 µm I.D. x 15 cm, 3 µm particle size, 100 Å pore size, Dionex, Sunnyvale, CA) and eluted at a flow rate of 300 nL/min using the following gradient: 3% solvent B in A (from 0-13 min), 3-7% solvent B in A (from 13-16 min), 7-25% solvent B in A (from 16-48 min), 25-40% solvent B in A (from 48-65 min), 40-90% solvent B in A (from 65-75 min) and 90% solvent B in A (from 75-85 min), with a total runtime of 120 min including mobile phase equilibration. Solvents were prepared as follows: mobile phase A: 2% acetonitrile/98% of 0.1% formic acid (v/v) in water, and mobile phase B: 98% acetonitrile/2% of 0.1% formic acid (v/v) in water. Mass spectra and tandem mass spectra were recorded in positive-ion and “high-sensitivity” mode with a resolution of ~35,000 full-width half-maximum. The nanospray needle voltage was typically 2,400 V in HPLC-MS mode. After acquisition of ~ 3 samples, TOF MS spectra and TOF MS/MS spectra were automatically calibrated during dynamic LC-MS & MS/MS autocalibration acquisitions injecting 25 fmol beta-galactosidase. Automatic re-calibration of TOF-MS and TOF-MS/MS scans (after every 3 samples) guaranteed reliably high mass accuracy and MS detector precision over an extended period of time (weeks) without interruption of the HPLC-MS/MS acquisitions. Two different mass spectrometric acquisition workflows were performed in this study: **1) Data dependent acquisitions on the TripleTOF 5600**: for collision induced dissociation tandem mass spectrometry (CID-MS/MS), the mass window for precursor ion selection of the quadrupole mass analyzer was set to ± 1 *m/z*. The precursor ions were fragmented in a collision cell using nitrogen as the collision gas. Advanced information dependent acquisition (IDA) was used for MS/MS collection on the TripleTOF 5600 to obtain MS/MS spectra for the 30 most abundant parent ions following each survey MS1 scan (250 msec acquisition time per MS1 scan, and typically 50 msec acquisition time per each MS/MS). Dynamic exclusion features were based on value M not *m/z* and were set to an exclusion mass width 50 mDa and an exclusion duration of 15-20 sec. **2) Data independent acquisitions on the TripleTOF 5600**: SWATH MS2 acquisitions. In the SWATH MS2 acquisition, instead of the Q1 quadrupole transmitting a narrow mass range through to the collision cell, a wider window of ~25 *m/z* is passed in incremental steps over the full mass range (*m/z* 400-1000 with 24 SWATH segments, 80 msec accumulation time each, yielding a cycle time of 2.2 sec which includes one MS1 scan with 250 msec accumulation time). SWATH MS2 produces complex MS/MS spectra which are a composite of all the analytes within each selected Q1 *m/z* window.

*Bioinformatic database searches for TripleTOF 5600 -* In this study, mass spectral data sets were analyzed and searched with a Mascot [Pappin, D. J. et al. (1993) *Curr Biol* **3**, 327-332] server version 2.3.02 using SwissProt 2011_08 (27 July. 2011) protein database with a total number of 531,473 sequences and the species restriction set to *H. sapiens* (searching 20,245 human sequences). Search parameters for trypsin: 4 missed cleavages and specificity as C-terminal cleavage at lysine and arginine; Asp-N: 4 missed cleavages and specificity as N-terminal cleavage at aspartic and glutamic acid; Chymotrypsin: No enzyme specificity; Tryps+Asp-N: 4 missed cleavages and specificity as C-terminal cleavage at lysine and arginine or N-terminal cleavage at aspartic and glutamic acid. Variable modifications for all enzyme conditions included lys acetylation, Protein N-terminal acetylation, met oxidation, conversion of glu to pyroglutamic acid, deamidation of asparagines, phosphorylated Ser, Thr, Tyr, and lys ubiquitination. N-ethylmaleimide cysteine was set as a fixed modification, and precursor ion mass accuracy was set to < 40 ppm and fragment ion mass tolerances were set to 0.2 Da. The maximum expectation value for accepting individual peptide ion scores [-10*Log(*p*)] was set to ≤0.05, where *p* is the probability that the observed match is a random event. For all Mascot searches automatic decoy searches were performed choosing the *Decoy* checkbox within the search engine. For all further data processing peptide expectation values (e-values) lower than 0.05 (Trypsin=0.045, Asp-N=0.016, chymotrypsin=0.006, trypsin+Asp-N=0.02) were chosen to keep the FDR rates at 0.05 for all enzyme conditions.

Mass spectrometric data was also analyzed using the database search engine ProteinPilot [Shilov, I. V. et al. (2007) *Mol Cell Proteomics* **6**, 1638-1655] (AB SCIEX Beta 4.1.46, revision 460) using the Paragon algorithm (4.0.0.0, 459). The following sample parameters were used: trypsin digestion, cysteine alkylation set to N-ethylmaleimide, acetylation and phosphorylation emphasis, and species *H. sapiens*. Trypsin, Asp-N, and chymotrypsin enzymes were the default settings. Processing parameters were set to "Biological modification" and a thorough ID search effort was used. During the search, Protein Pilot performs an automatic mass recalibration of the data sets based on highly confident peptide spectra. Specifically, a first search iteration is done to select high confidence peptide identifications that are used to recalibrate both the MS and MS/MS data, which is automatically re-searched. All data files were searched using the SwissProt 2011_08 (27 Jul. 2011) with a total of 531,473 sequences (20,245 human protein sequences). For Protein Pilot Searches, to assess and restrict rates of false positive peptide/protein identifications, we used the Proteomics System Performance Evaluation Pipeline (PSPEP) tool available in ProteinPilot 4.0 and 4.1 beta, respectively. This tool automatically creates a concatenated forward and reverse decoy database, and provides an Excel output of the experimentally determined false discovery rate at the spectral, peptide and protein levels with standard statistical errors [Shilov, I. V. et al. (2007) *Mol Cell Proteomics* **6**, 1638-1655]. The discriminating variable for the Paragon Algorithm is the peptide confidence value, which is a 0-99 scaled real number [Shilov, I. V. et al. (2007) *Mol Cell Proteomics* **6**, 1638-1655]. For database searches, a cut-off peptide confidence value of 99 was chosen with the following justification. For searching the databases the Protein Pilot false discovery rate (FDR) analysis tool (PSPEP) algorithm [Shilov, I. V. et al. (2007) *Mol Cell Proteomics* **6**, 1638-1655] provided, at a minimum, a global FDR of 1% and a local FDR at 5% in all cases.
